# Improvement in the Tracking Performance of a Maneuvering Target in the Presence of Clutter

**DOI:** 10.3390/s22207848

**Published:** 2022-10-16

**Authors:** Ghawas Ali Shah, Sumair Khan, Sufyan Ali Memon, Mohsin Shahzad, Zahid Mahmood, Uzair Khan

**Affiliations:** 1 Department of Electrical and Computer Engineering, Abbottabad Campus, COMSATS University Islamabad, Abbottabad 22060, Pakistan; 2 Department of Computer Science, Abbottabad Campus, COMSATS University Islamabad, Abbottabad 22060, Pakistan; 3 Department Defense System Engineering, Sejong University, Seoul 05006, Korea

**Keywords:** IPDA, IMM, IMM-IPDA, fixed lag smoothing, RMSE, TTR, mode probabilities, cluttered environment

## Abstract

The proposed work uses fixed lag smoothing on the interactive multiple model-integrated probabilistic data association algorithm (IMM-IPDA) to enhance its performance. This approach makes use of the advantages of the fixed lag smoothing algorithm to track the motion of a maneuvering target while it is surrounded by clutter. The suggested method provides a new mathematical foundation in terms of smoothing for mode probabilities in addition to the target trajectory state and target existence state by including the smoothing advantages. The suggested fixed lag smoothing IMM-IPDA (FLs IMM-IPDA) method’s root mean square error (RMSE), true track rate (TTR), and mode probabilities are compared to those of other recent algorithms in the literature in this study. The results clearly show that the proposed algorithm outperformed the already-known methods in the literature in terms of these above parameters of interest.

## 1. Introduction

Target tracking in the presence of clutter has received much attention in recent times due to proposing improvements in the tracking algorithms. The tracking procedure should incorporate different problems while tracking a moving target in the presence of clutter [1]. Among these problems, tracking a maneuvering target in a highly cluttered environment has received a lot of attention due to its practicality in real tracking environments [2,3,4,5,6,7]. In essence, the target going through a maneuver diverges from the assumption of a constant velocity constraint set for the moving targets in different tracking algorithms [8,9,10,11]. This makes the tracking performance more compromised under difficult tracking conditions such as high clutter density in the tracking environment.

A number of single target non-maneuvering tracking algorithms are available in the literature, which can track a target moving without any maneuver during their movement [12,13,14], but these algorithms do not provide any procedure to track the maneuvering target with accuracy. Some authors have used multi-scan single target tracking algorithms to achieve the tracking accuracy for maneuvering targets [15,16,17,18,19,20], but at the end they have increased the complexity of the algorithm without providing a new mathematical structure for the given problem. Multi-target tracking algorithms are also used in the literature to address the maneuvering target tracking issue. These algorithms are inherently mathematically complex and time consuming [21,22,23,24,25].

The maneuvering targets are tracked more efficiently by using multiple model tracking approaches such as the interacting multiple model (IMM) tracking algorithm. In Ref. [26], the author used a combination of the interacting multiple model (IMM) and modified gain extended Kalman filter to obtain IMM-MGEKF algorithm for improvement in the performance of IMM-based algorithms. In Ref. [27], the author used the IMM–STSRCKF algorithm (which is an extension of the IMM algorithm using the curvature Kalman filter) to track the target that undergoes maneuvering. Multi sensor information is fused and then incorporated in IMM and PDF tracking algorithms for maneuvering target tracking in Ref. [28]. By using multiple models and target existence state information, an interacting multiple model-integrated probabilistic data association IMM-IPDA algorithm is proposed in Ref. [29]. It shows the improvement in the performance of tracking algorithm in terms of target hybrid state.

Smoothing algorithms are used in a number of applications in target tracking algorithms to improve their performance. The smoothing algorithms increase the computational time and tend to offer delays in any tracking algorithm [12]. However with smoothing, a more accurate and reliable picture of the environment can be achieved. The augmented smoothing algorithm improves the RMSE performance of the existing tracking algorithms but has not provided the complete mathematical structure of the algorithm [30,31]. The FLs-IPDA provides a formula to improve both the target trajectory state and target existence state by using all possible existence events [32]. An extension of the same work with more general analogy is explained in Ref. [33]. In Ref. [34], the authors have used the forward-backward prediction model for smoothing, but they have not provided any mathematical framework for smoothed mode probabilities. All these smoothing algorithms have not studied the status of hybrid target state during maneuvering in the presence of clutter.

In this work, a fixed lag smoothing algorithm is devised for the IMM-IPDA algorithm to improve the performance of the IMM-IPDA algorithm in a cluttered environment. The main contributions of proposed study are:

- This work has enhanced the earlier work proposed in Ref. [33] by adding novelty in terms of mathematical modeling for maneuvering target tracking and its fixed lag smoothing;
- Mathematical formulation for the FLs IMM-IPDA in terms of smoothed target trajectory state estimation, smoothed target existence state update, and smoothed mode probabilities;
- Utilization of the fixed lag smoothing algorithm to improve the tracking performance of IMM-IPDA;
- Improvement in the RMSE, TTR, and mode probabilities using the fixed lag smoothing algorithm;
- A complete set of simulations are performed in MATLAB to prove the above contributions.

This paper is divided into different sections to properly describe the principal of proposed work. The target motion model and related concepts are addressed in Section 2. The mathematical model and different derivations are provided in Section 3. In Section 4, complete analyses and a discussion of the results are made. The conclusion is provided at the end to summarize the proposed work.

## 2. Mathematical Model

In this work, the target is assumed to switch in-between the target existence and non-existence state randomly during its motion. It is assumed that the target follows the Markov chain model for its propagation. The probability of the target existence is defined as

(1) $${\bar{p}}_{11}\equiv P\left\{\chi_{k}|\chi_{k-1}\right\}\approx 1-\frac{\Delta T_{k-1,k}}{T_{avg}},$$

where $\Delta T_{k-1,k}$ defines the time interval in-between scan k−1 and *k*. $T_{avg}$ is the average duration of the target existence. ${\bar{p}}_{11}$ is the probability that the target exists at any current scan *k* conditioned on its existence at scan k−1. The target existence ($\chi_{k}$) and its non-existence (${\bar{\chi }}_{k}$) are mutually exclusive and exhaustive events. Thus, the total probability sum is 1 at any particular scan.

(2) $$P\left\{\chi_{k}\right\}+P\left\{{\bar{\chi }}_{k}\right\}=1.$$

The target follows the state transition model defined in (3).

(3) $$x_{k}=Fx_{k-1}+w_{k},$$

where $w_{k}$ is assumed to be a zero mean white Gaussian noise with known covariance Q. It represents the uncertainty in the assumed target motion model (3). Q is defined as

(4) $$Q=q\left[\begin{array}{cc} 0.25T^{4}I_{m} & 0.5T^{3}I_{m}\\ 0.5T^{3}I_{m} & T^{2}I_{m} \end{array}\right],$$

where *q* is the plant noise parameter, *m* is the dimension of measurement vector. The Q matrix becomes n− dimensional in (4). In (3), $x_{k}$ is the target trajectory state at scan *k* and it is defined as

(5) $$x_{k}=\left[\begin{array}{c} x_{k}\\ {\dot{x}}_{k}\\ y_{k}\\ {\dot{y}}_{k} \end{array}\right],$$

where $x_{k}$ and $y_{k}$ are the target position coordinates in Cartesian coordinates, while ${\dot{x}}_{k}$ and ${\dot{y}}_{k}$ are the respective velocity components of the target.

### 2.1. Measurement Model

The measurements from the target at any scan *k* are defined as

(6) $$y_{k}=Hx_{k}+v_{k},$$

where H is the measurement matrix, $y_{k}$ is the measurement received at any current scan *k*, and $v_{k}$ is the measurement noise, which is assumed to be zero mean white Gaussian noise. Its covariance **R** is assumed to be

(7) $$R_{k}=\left[\begin{array}{cc} \sigma_{xx}^{2} & 0\\ 0 & \sigma_{yy}^{2} \end{array}\right],$$

where $\sigma_{xx}$, $\sigma_{yy}$ are the variance in the *x*-axis and *y*-axis, respectively, and are assumed to 5 m in this study equally. In (6), **H** is defined as

(8) $$H=\left[\begin{array}{cc} I_{m} & 0_{m} \end{array}\right],$$

where $I_{m}$ is the identity matrix of measurement order *m* and $0_{m}$ is the matrix of zeroes with dimension *m*.

### 2.2. Target Motion Models

In this study, it is assumed that the target performs maneuvering during its motion. Hence, the target will have two possible motion models to form its trajectory. One is the constant velocity model (CV), while the other one is the constant acceleration model (CA). For the CA model, the state trajectory is

(9) $$x_{k}=\;\left[\begin{array}{c} x_{k}\\ {\dot{x}}_{k}\\ {\ddot{x}}_{k}\\ y_{k}\\ {\dot{y}}_{k}\\ {\ddot{y}}_{k} \end{array}\right],$$

The variable (x,y) represents target position, $\left(\dot{x},\dot{y}\right)$ denotes the velocity of target and $\left(\ddot{x},\ddot{y}\right)$ represents the acceleration of the target. For the CV model, the acceleration components in the state trajectory (9) becomes 0, and hence the trajectory state vector for the CV model will become the same as given in (5). The CV state transition matrix $F_{v}$ is

(10) $$F_{v}=\left[\begin{array}{cc} f_{v} & 0_{3\times 3}\\ 0_{3\times 3} & f_{v} \end{array}\right],$$

where parameter $f_{v}$ is a three-dimensional matrix given as

(11) $$f_{v}=\left[\begin{array}{ccc} 1 & T & 0\\ 0 & 1 & 0\\ 0 & 0 & 0 \end{array}\right]$$

where *T* is the sampling time. On the other hand, error covariance matrix $Q_{v}$ is

(12) $$Q_{v}=q\left[\begin{array}{cc} q_{v} & 0_{3\times 3}\\ 0_{3\times 3} & q_{v} \end{array}\right],$$

where *q* denotes the plant noise parameter and $q_{v}$ is a three-dimensional matrix

(13) $$q_{v}=\left[\begin{array}{ccc} 0.33T^{3} & 0.5T^{2} & 0\\ 0.5T^{2} & 0 & 0\\ 0 & 0 & 0 \end{array}\right].$$

The state transition matrix $F_{a}$ for the CA model is defined as

(14) $$F_{a}=\left[\begin{array}{cc} f_{a} & 0_{3\times 3}\\ 0_{3\times 3} & f_{a} \end{array}\right],$$

where the three-dimensional matrix $f_{a}$ is the state transition matrix and is equal to

(15) fa=1TT2T22201T001.

The error covariance matrix $Q_{a}$ for CA model is

(16) $$Q_{a}=q\left[\begin{array}{cc} q_{a} & 0_{3\times 3}\\ 0_{3\times 3} & q_{v} \end{array}\right],$$

where $q_{v}$ is defined in (13) and $q_{a}$ is defined as

(17) qa=T5T52020T4T488T3T366T4T488T5T52020T3T388T5T566T4T488T5T52020.

For constant acceleration state vector (9), the H matrix will have an additional *m*-dimensional zeros column.

## 3. Fixed Lag Smoothing IMM IPDA

In this section, a step-by-step approach towards the working of the proposed algorithm is provided. Tracks are initialized using the two-point difference [12] method. By considering the target birth as a random event, this test is repeated at every scan for tracks initialization. At any scan, the target starts with a two point initialization step.

Tracks are initialized at any scan *k* with target initial augmented trajectory state for the *j*th model as

(18) $${\hat{x}}_{k_{0}|k_{0}}^{A,j}={\left[{\hat{x}}_{k_{0}|k_{0}}^{j}{\hat{x}}_{k_{0}-1|k_{0}}^{j}...{\hat{x}}_{k_{0}-N|k_{0}}^{j}\right]}^{T},$$

and its associated covariance is initialized as

(19) $$P_{k_{0}|k_{0}}^{A,j}=\left[\begin{array}{cccc} P_{k_{0}|k_{0}}^{j} & 0_{n\times n} & \cdots & 0_{n\times n}\\ 0_{n\times n} & P_{k_{0}-1|k_{0}}^{j} & \cdots & 0_{n\times n}\\ \vdots & \ddots & \cdots & \vdots \\ 0_{n\times n} & 0_{n\times n} & \cdots & P_{k_{0}-N|k_{0}}^{j} \end{array}\right].$$

$k_{0}$ in the subscript denotes the initial state of the target. ’*A*’ in the superscript is the significance of the augmented state while the *j* denotes the *j*th model in progress.

The augmented state transition matrix $F_{k}^{A,j}$ at any scan *k* for constant velocity and constant acceleration model are defined as

(20) $$F^{A,j}=\left[\begin{array}{cccc} F_{v/a} & 0_{n\times n} & \cdots & 0_{n\times n}\\ I_{n\times n} & 0_{n\times n} & \cdots & 0_{n\times n}\\ 0_{n\times n} & I_{n\times n} & \cdots & 0_{n\times n}\\ \vdots & \vdots & \ddots & \vdots \\ 0_{n\times n} & 0_{n\times n} & \cdots & I_{n\times n} \end{array}\right],$$

where $F_{v/a}$ is the definition of the state transition matrix defined in (10) and (14) for CV and CA models.

The process noise covariance matrix $Q_{k}^{A,j}$ is

(21) $$Q^{A,j}=\left[\begin{array}{cccc} Q_{v/a} & 0_{n\times n} & \cdots & 0_{n\times n}\\ 0_{n\times n} & 0_{n\times n} & 0_{n\times n} & 0_{n\times n}\\ \vdots & 0_{n} & \ddots & \vdots \\ 0_{n\times n} & 0_{n\times n} & \cdots & I_{n\times n} \end{array}\right],$$

where $Q_{v/a}$ is the process noise covariance matrix conditioned on (12) and (16).

### Track Information Mixing

The mixing step is key to the IMM initialization at any current scan *k*. For any scan *k*, it is assumed that the transition probability, which defines the switching from mode (model) to mode (model), is already known. The mode transition probabilities can be modeled in the form of matrix

(22) $$p_{ij}=\left[\begin{array}{cc} 0.98 & 0.02\\ 0.02 & 0.98 \end{array}\right].$$

The selection procedure for the values in (22) is discussed in Ref. [32]. The sum of these mode transition probabilities is always equal to 1.

(23) $$\sum_{j=1}^{r}p_{ij}=1$$

The mixing steps in IMM algorithm are summarized below:

**1: Mixing mode probability**

(24) $$\mu_{k-1|k-1}^{i|j}=P\left\{M_{k-1}^{i}|M_{k}^{j},Y^{k-1}\right\},$$

(25) $$\mu_{k-1|k-1}^{i|j}=\frac{p_{ij}\mu_{k-1|k-1}^{i}}{\sum_{i=1}^{r}p_{ij}\mu_{k-1|k-1}^{i}}.$$

The *i* and *j* represents the previous and current scans index, respectively. $\mu_{k-1|k-1}^{i}$ is the mode probability at scan k−1. $M_{k}^{j}$ is the target motion model at *j*th scan, while $M_{k-1}^{i}$ is the target motion model in *i*th scan. The denominator in (25) is the normalization term.

**2: Mixing target trajectory state and error covariance matrix subject to initial conditions**

(26) $${\hat{x}}_{k-1|k-1}^{A,0j}=\sum_{i=1}^{r}\left[\begin{array}{c} p\left({\hat{x}}_{k}^{A}|M_{k-1}^{i},M_{k}^{j},\chi_{k},Y^{k-1}\right)P\left\{M_{k-1}^{i}|M_{k}^{j},Y^{k-1}\right\} \end{array}\right],$$

(27) $${\hat{x}}_{k-1|k-1}^{A,0j}=\sum_{i=1}^{r}{\hat{x}}_{k-1|k-1}^{A_{j}}\mu_{k-1|k-1}^{i|j},$$

where ${\hat{x}}_{k-1|k-1}^{A_{j}}$ is target trajectory state estimate at scan k−1 conditioned on *j*th mode. The mixing state error covariance is

(28) $$P_{k-1|k-1}^{A,0j}=\sum_{i=1}^{r}\mu_{k-1|k-1}^{i|j}\left(\begin{array}{c} P_{k-1|k-1}^{A,j}+\left[\left(A_{x}\right){\left(A_{x}\right)}^{T}\right] \end{array}\right)$$

where

(29) $$A_{x}={\hat{x}}_{k-1|k-1}^{A,i}-{\hat{x}}_{k-1|k-1}^{A,oj}$$

$P_{k-1|k-1}^{A,j}$ is state error covariance update at scan k−1 for *j*th model.

**3: Prediction Process**

With the use of results summarized in (27) and (28), the target trajectory state and its associated error covariance are predicted for the current scan *k* as

(30) $${\hat{x}}_{k|k-1}^{A,j}=F^{A,j}{\hat{x}}_{k-1|k-1}^{A,0j},$$

and

(31) $$P_{k|k-1}^{A,j}=F^{A,j}P_{k-1|k-1}^{A,0j}{\left(F^{A,j}\right)}^{T}+Q^{A,j}.$$

All parameters used in (30) and (31) are defined in the above sections.

**4: Update Process**

In this step, the mode probability, target trajectory state and its associated error covariance, and the target existence state is updated using each *r*th measurement information $y_{k}\left(r\right)$ from the measurement vector $y_{k}$ received at current scan *k*. The measurement selection is carried out using the following selection criteria

(32) $${\left(\;y_{k}\left(r\right)-H^{A}{\hat{x}}_{k|k-1}^{A,j}\right)}^{T}\;{\left(S_{k}^{A,j}\right)}^{-1}\;\left(\;y_{k}\left(r\right)-H^{A}{\hat{x}}_{k|k-1}^{A,j}\right)\leq g,$$

where *g* is the gating threshold. Its value is selected as the 3σ limit on standard deviation. $S_{k}^{A,j}$ is defined as

(33) $$S_{k}^{A,j}=H^{A}P_{k|k-1}^{A,j}{\left(H^{A}\right)}^{T}+R.$$

The measurements from the measurement set $y_{k}$, which satisfies the criteria defined in Equation (32), are selected further for the estimation process.

**4.1: Calculation of data association probabilities at the current scan**

Each measurement $y_{k}\left(r\right)$ at the current scan is associated with the target of interest and its association probability β is calculated for both the null hypothesis (none of the measurement belongs to the target) and the other hypothesis [12].

(34) $$\beta_{k|k}\left(r,M^{j}\right)=\frac{1}{\Delta_{k}\left(M^{j}\right)}\left\{\begin{array}{cc} 1-P_{d}P_{g}; & r=0\\ \frac{P_{d}P_{g}p_{k}\left(r,M^{j}\right)}{\rho }; & r>0 \end{array}\right.,$$

where r=0 implies the null hypothesis and r>0 implies the detection hypothesis. $P_{d}$ is the detection probability and the $P_{g}$ is the gating probability. The values of these parameters are selected with the assumption that if the target is detected then it is certain that it will lie in the validation gate. ρ is the clutter density. The likelihood function for the measurement $y_{k}\left(r\right)$ conditioned on model $M^{j}$ is

(35) $$p_{k}\left(r,M^{j}\right)=\frac{1}{\left|\sqrt{2\pi S_{k}^{A,j}}\right|}\exp^{\left(-\frac{1}{2}{\left[y_{k}\left(r\right)-H^{A}{\hat{x}}_{k|k-1}^{A,j}\right]}^{T}{\left(S_{k}^{A,j}\right)}^{-1}\left[y_{k}\left(r\right)H^{A}{\hat{x}}_{k|k-1}^{A,j}\right]\right).}$$

The $\Delta_{k}\left(M^{j}\right)$ is defined as

(36) Δk(Mj)=1−PdPg+PdPg∑r=1mkpkr,Mjρ

.

**4.2: Mode probability update at current scan**

The mode probability at the current scan for *j*th mode, conditioned on the *r*th measurement $y_{k}\left(r\right)$ is

(37) $$\begin{array}{c} P\left\{M_{k}^{j}\left(r\right)|Y^{k}\right\}=\mu_{k|k}\left(r,M^{j}\right),\\ \mu_{k|k}\left(r,M^{j}\right)=\frac{p_{k}\left(r,M^{j}\right)\mu_{k|k-1}\left(M^{j}\right)}{\sum_{m=1}^{M}p_{k}\left(r,M^{m}\right)\mu_{k|k-1}\left(M^{m}\right)} \end{array},$$

where

(38) $$P\left\{M_{k}^{j}|Y^{k-1}\right\}\;=\mu_{k|k-1}\left(M^{j}\right)=\;\sum_{m=1}^{M}p_{mj}\mu_{k-1|k-1}\left(M^{m}\right).$$

For the non-detection event r=0, (37) will be defined as

(39) $$\mu_{k|k}\left(0,M^{j}\right)=\mu_{k|k-1}\left(M^{j}\right).$$

To obtain the updated mode probability for the *j*th mode at current scan, we have to use (37) for all measurements to the mode hypothesis as

(40) $$P\left\{M_{k}^{j}|Y^{k}\right\}=\mu_{k|k}\left(M^{j}\right)=\sum_{r=0}^{m_{k}}\beta_{k|k}\left(r\right)\mu_{k|k}\left(r,M^{j}\right),$$

where

(41) $$\beta_{k|k}\left(r\right)=\frac{1}{\Delta_{k}}\left\{\begin{array}{cc} 1-P_{d}P_{g}; & r=0\\ P_{d}P_{g}\frac{p_{k}\left(r\right)}{\rho }; & r>0 \end{array}\right.$$

In (41), the measurement likelihood $p_{k}\left(r\right)$ is

(42) $$p_{k}\left(r\right)=\sum_{m=1}^{M}\mu_{k|k-1}\left(M^{m}\right)p_{k}\left(r,M^{m}\right)$$

and

(43) Δk=1−PdPg+PdPg∑r=1mkpkrρ

**4.3: Estimation of the trajectory state and error covariance at the current scan**

The target trajectory state estimate at scan *k* is update conditioned on the *r*th measurement and *j*th model as

(44) $${\hat{x}}_{k|k}^{A,j}\left(r\right)=x_{k|k-1}^{A,j}+K_{k}^{A,j}\left(y_{k}\left(r\right)-{\hat{y}}_{k|k-1}^{j}\right),$$

where

(45) $${\hat{y}}_{k|k-1}^{j}=H^{A}{\hat{x}}_{k|k-1}^{A,j},$$

and the Kalman gain at current scan *k* is

(46) $$K_{k}^{A,j}=P_{k|k-1}^{A,j}{\left(H^{A}\right)}^{T}{\left(S_{k}^{A,j}\right)}^{-1}.$$

The measurement innovation covariance matrix $S_{k}^{A,j}$ is defined as

(47) $$S_{k}^{A,j}=H^{A}P_{k|k-1}^{A,j}{\left(H^{A}\right)}^{T}+R,$$

where

(48) $$H^{A}=\left[\begin{array}{cccc} H & 0_{m,n} & .... & 0_{m,n} \end{array}\right].$$

The updated trajectory state error covariance matrix is

(49) $$P_{k|k}^{A,j}\left(r\right)=P_{k|k-1}^{A,j}+K_{k}^{A,j}H^{A}P_{k|k-1}^{A,j}.$$

The target trajectory state update at the current scan conditioned on all models and measurement associations is

(50) $${\hat{x}}_{k|k}^{A}=\sum_{r=0}^{m_{k}}\sum_{m=1}^{M}{\hat{x}}_{k|k}^{A,m}\left(r\right)\beta_{k|k}\left(r,M^{m}\right)\mu_{k|k}\left(r,M^{m}\right),$$

where for the r=0 and *m*th model

(51) $${\hat{x}}_{k|k}^{A,m}\left(0\right)=x_{k|k-1}^{A,m}.$$

Other parameters are defined in the above set of equations. The associated state error covariance matrix is calculated as

(52) $$P_{k|k}^{A}=\sum_{r=0}^{m_{k}}\sum_{m=1}^{M}\begin{array}{c} \mu_{k|k}\left(r,M^{m}\right)\beta_{k|k}\left(r,M^{m}\right)\left\{P_{k|k}^{A,m}\left(r\right)+\left[Df\right]{\left[Df\right]}^{T}\right\} \end{array},$$

where

(53) $$Df=\left[{\hat{x}}_{k|k}^{A,m}\left(r\right)-{\hat{x}}_{k|k}^{A}\right].$$

**4.4: Target existence state update at the current scan**

The target existence state $\chi_{k}$ at the current scan *k* is also updated for the maneuvering target as

(54) $$\begin{array}{c} P\left\{\chi_{k}|Y^{k}\right\}=\frac{p\left(y_{k}|\chi_{k},Y^{k-1}\right)}{p\left(y_{k}|Y^{k-1}\right)}P\left\{\chi_{k}|Y^{k-1}\right\}.\\ \;\;\;\;\;\;\;\;\;\;\;\;\;\;\;\;\;\;\;\;=\frac{\Delta_{k}P\left\{\chi_{k}|Y^{k-1}\right\}}{1-\left(1-\Delta_{k}\right)P\left\{\chi_{k}|Y^{k-1}\right\}}, \end{array}$$

where

(55) $$\begin{array}{c} P\left\{\chi_{k}|Y^{k-1}\right\}=P\left\{\chi_{k}|\chi_{k-1}\right\}P\left\{\chi_{k-1}|Y^{k-1}\right\}.\\ \;\;\;\;\;\;\;\;\;\;\;\;\;\;\;\;\;\;\;\;\;\;\;\;\;\;\;\;={\bar{p}}_{11}P\left\{\chi_{k-1}|Y^{k-1}\right\}. \end{array}$$

The ${\bar{p}}_{11}$ is defined in (1), and $P\left\{\chi_{k-1}|Y^{k-1}\right\}$ is the track existence state at scan k−1. The parameter $\Delta_{k}$ is defined in (43).

**5: Smoothed State Update**

The smoothing of the target trajectory state and target existence state at fixed lag *N* is carried out in this section. The smoothing principle proposed in Ref. [33] is used here to obtain the smoothed hybrid state at fixed lag *N*. The authors have used the fixed lag smoothing algorithm on the integrated track splitting filter to obtain the smoothed target trajectory state and target existence state at the same time. In this work, the smoothing principle is carried out for the maneuvering target scenario. In addition to the improvement in the target hybrid state, the mode weights are also smoothed at fixed lag *N*.

**5.1: Augmented Smoothed Target Trajectory State Update**

At each time step *k*, we also obtain the augmented target trajectory state conditioned on *j*th model as

(56) $${\hat{x}}_{k|k}^{A,j}=\sum_{r=0}^{m_{k}}{\hat{x}}_{k|k}^{A,j}\left(r\right)\beta_{k|k}\left(r,M^{j}\right).$$

**Figure 1:**
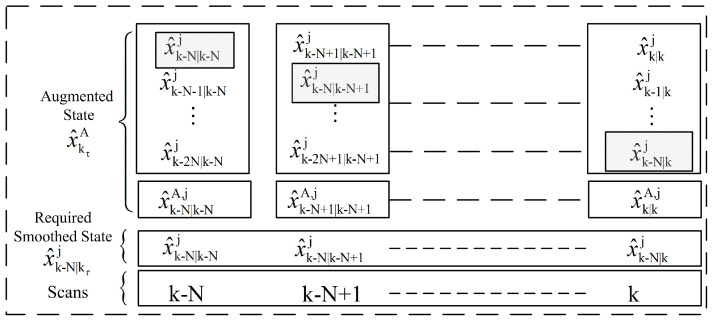
Smoothing window overview.

The augmented state Equation (56) also provides the smoothed state vector at lag k−N using the measurement information available until the current scan *k*. This idea is presented in Figure 1 for a smoothing window of lag size *N*. At each scan we need to collect the smoothed state ${\hat{x}}_{k-N|k_{\tau }}^{j}$, where $k_{\tau }$ represents the index in the smoothing window. In Section 5.2, the result of (56) will be used to obtain the smoothed target trajectory state at fixed lag *N*.

**5.2: Smoothed Target Existence State Update**

For the smoothed target existence state $\chi_{k-N}$ under maneuvering at fixed lag *N*, the final results of Ref. [33] are used here. Let $k_{\tau }$ be the running index in the smoothing window and is defined as $k_{\tau }=k-N+w$, where 0≤w≤N. The smoothed target existence state for w=0 is

(57) $$P\left\{\chi_{k_{\tau }}|Y^{k}\right\}=\frac{T_{1}+\left[\sum_{s=1}^{N-w}\left[\prod_{n=s-1}^{N-1}\Lambda_{k-n}\right]\Omega_{k_{\tau }}\left(s\right)\right]}{Den(\Omega_{k_{\tau }})}\;\;$$

where

(58) $$T_{1}=\left(1-p_{11}\right)P\left\{\chi_{k-N}|Y^{k-N}\right\}.$$

For 1≤w≤N

(59) $$P\left\{\chi_{k_{\tau }}|Y^{k}\right\}=\frac{\sum_{s=1}^{N-w+1}\left[\prod_{n=s-1}^{N-1}\Lambda_{k-n}\right]\Omega_{k_{\tau }}\left(s\right)}{Den(\Omega_{k_{\tau }})}\;\;,$$

where

(60) $$\begin{array}{c} Den\left(\Omega \right)=1-\left(p_{11}P\left\{X_{k-N}|Y^{k-N}\right\}\right)+\left[\sum_{s=1}^{N}\left[\prod_{n=s-1}^{N-1}\Lambda_{k-n}\right]\Omega_{k_{\tau }}\left(s\right)\right] \end{array},$$

and

(61) $$\Omega_{k_{\tau }}\;\left(s\right)\;=\;\left\{\;\begin{array}{c} P\left\{\chi_{k}|Y^{k-N}\right\}\;;s=1\\ P\left\{\chi_{k-s+1}|Y^{k-N}\right\}\;-\;P\left\{\chi_{k-s+2}|Y^{k-N}\right\};s>1 \end{array}\right.$$

**5.3: Smoothed Mode Probability Update**

**Figure 2:**
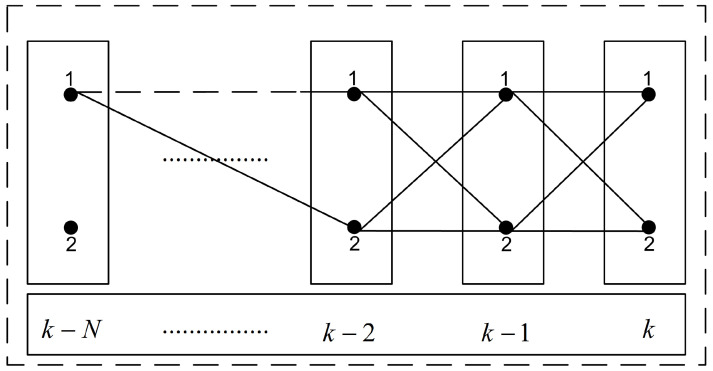
Joint mode events in the smoothing window (lag: *N*, modes: 2).

**Figure 3:**
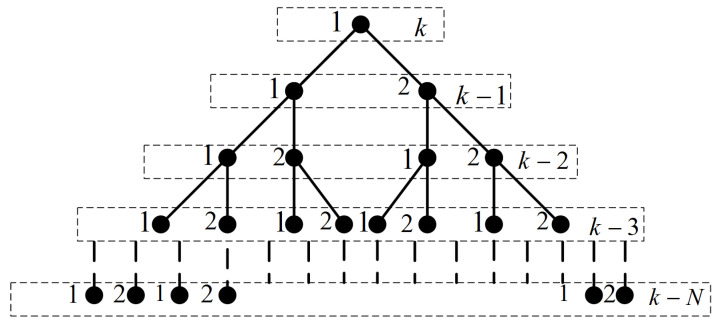
Joint mode events in the smoothing window for M = 2.

The mode probabilities are also smoothed in the proposed algorithm at fixed lag *N*. The mode probabilities are smoothed at fixed lag *N* for each *j*th model by assuming all possible joint events with respect to the motion models at each scan in the smoothing window. This principle is illustrated in Figure 2, where M=2 is observed as a special case. For clarity it is assumed that $M^{1}$ at scan k−N is to be smoothed. The same procedure will be repeated for any *j*th model $M^{j}$. In Figure 3, a tree diagram is presented for the set of possible hypothesis in a smoothing window of lag size *N* and M=2. It is presented for any single *j*th mode (in Figure 3, j=1) at scan *k* taken as reference and its backward propagation in the smoothing window until scan k−N is observed. In general, the number of possible joint events from scan *k* to scan k−N grows as a function $M^{N+1}$.

Here, to begin with, a special case of lag size N=2 and number of motion models M=2 is considered. Later in the section, the results will be generalized for any lag *N* and for any number of motion models. The smoothed mode probability for any $M^{1}$ model at scan k−2 is calculated as

(62) $$\begin{array}{c} P\left\{M_{k-2}^{1}|Y^{k}\right\}=P\left\{M_{k-2}^{1},M_{k-1}^{1},M_{k}^{1}|Y^{k}\right\}+P\left\{M_{k-2}^{1},M_{k-1}^{1},M_{k}^{2}|Y^{k}\right\}\\ \;+P\left\{M_{k-2}^{1},M_{k-1}^{2},M_{k}^{1}|Y^{k}\right\}\;+\;P\left\{M_{k-2}^{1},M_{k-1}^{2},M_{k}^{2}|Y^{k}\right\}\;. \end{array}$$

The first term on the right side of equality in (62) is defined as

(63) $$\begin{array}{c} P\left\{M_{k-2}^{1},M_{k-1}^{1},M_{k}^{1}|Y^{k}\right\}=\frac{p\left(y_{k},y_{k-1}|M_{k-2}^{1},M_{k-1}^{1},M_{k}^{1},Y^{k-2}\right)}{p\left(y_{k},y_{k-1}|Y^{k-2}\right)}P_{111} \end{array},$$

where

(64) $$P_{111}=P\left\{M_{k-2}^{1},M_{k-1}^{1},M_{k}^{1}|Y^{k-2}\right\},$$

(65) $$P_{111}=p_{11}^{2}P\left\{M_{k-2}^{1}|Y^{k-2}\right\},$$

where, $p_{11}$ is defined in (22). $P\left\{M_{k-2}^{1}|Y^{k-2}\right\}$ is the estimated mode probability of $M^{1}$ at scan k−2, and it is obtained in (40). Similarly, the next three terms on the right hand side of (62) can be solved, such that the second term becomes

(66) $$\begin{array}{c} P\left\{M_{k-2}^{1},M_{k-1}^{1},M_{k}^{2}|Y^{k}\right\}=\frac{p\left(y_{k},y_{k-1}|M_{k-2}^{1},M_{k-1}^{1},M_{k}^{2},Y^{k-2}\right)}{p\left(y_{k},y_{k-1}|Y^{k-2}\right)}P_{112} \end{array},$$

where

(67) $$P_{112}=P\left\{M_{k-2}^{1},M_{k-1}^{1},M_{k}^{2}|Y^{k-2}\right\},$$

(68) $$P_{112}=p_{12}p_{11}P\left\{M_{k-2}^{1}|Y^{k-2}\right\}.$$

The third term is

(69) $$\begin{array}{c} P\left\{M_{k-2}^{1},M_{k-1}^{2},M_{k}^{1}|Y^{k}\right\}=\frac{p\left(y_{k},y_{k-1}|M_{k-2}^{1},M_{k-1}^{2},M_{k}^{1},Y^{k-2}\right)}{p\left(y_{k},y_{k-1}|Y^{k-2}\right)}P_{121} \end{array},$$

where

(70) $$P_{121}=P\left\{M_{k-2}^{1},M_{k-1}^{2},M_{k}^{1}|Y^{k-2}\right\},$$

(71) $$P_{121}=p_{21}p_{12}P\left\{M_{k-2}^{1}|Y^{k-2}\right\},$$

and the fourth term on the right hand side of (62) is

(72) $$\begin{array}{c} P\left\{M_{k-2}^{1},M_{k-1}^{2},M_{k}^{2}|Y^{k}\right\}=\frac{p\left(y_{k},y_{k-1}|M_{k-2}^{1},M_{k-1}^{2},M_{k}^{2},Y^{k-2}\right)}{p\left(y_{k},y_{k-1}|Y^{k-2}\right)}P_{122} \end{array},$$

where

(73) $$P_{122}=P\left\{M_{k-2}^{1},M_{k-1}^{2},M_{k}^{2}|Y^{k-2}\right\},$$

(74) $$P_{122}=p_{22}p_{12}P\left\{M_{k-2}^{1}|Y^{k-2}\right\}.$$

The derivations to obtain the results in Equations (65), (68), (71), and (74) are available in Appendix A. Using the Equations (63)–(73) and some previous results in (62) as

(75) $$\begin{array}{c} \;P\left\{M_{k-2}^{1}|Y^{k}\right\}\;=\;\frac{\Delta_{k}\left(M^{1}\right)\Delta_{k-1}\left(M^{1}\right)}{p\left(y_{k},y_{k-1}|Y^{k-2}\right)}p_{11}^{2}P\left\{M_{k-2}^{1}|Y^{k-2}\right\}\\ \;\;\;\;\;\;\;\;\;\;\;\;\;\;\;\;\;\;\;\;\;\;\;\;\;\;\;\;+\frac{\Delta_{k}\left(M^{2}\right)\Delta_{k-1}\left(M^{1}\right)}{p\left(y_{k},y_{k-1}|Y^{k-2}\right)}p_{12}p_{11}P\left\{M_{k-2}^{1}|Y^{k-2}\right\}\\ \;\;\;\;\;\;\;\;\;\;\;\;\;\;\;\;\;\;\;\;\;\;\;\;\;\;\;\;\;+\frac{\Delta_{k}\left(M^{1}\right)\Delta_{k-1}\left(M^{2}\right)}{p\left(y_{k},y_{k-1}|Y^{k-2}\right)}p_{21}p_{12}P\left\{M_{k-2}^{1}|Y^{k-2}\right\}\\ \;\;\;\;\;\;\;\;\;\;\;\;\;\;\;\;\;\;\;\;\;\;\;\;\;\;\;\;\;+\frac{\Delta_{k}\left(M^{2}\right)\Delta_{k-1}\left(M^{2}\right)}{p\left(y_{k},y_{k-1}|Y^{k-2}\right)}p_{22}p_{12}P\left\{M_{k-2}^{1}|Y^{k-2}\right\} \end{array}\;,$$

where $\Delta_{k-1}\left(M^{m}\right)$ and $\Delta_{k}\left(M^{m}\right)$ for m=1:2 are the likelihood ratios conditioned on models for scans k−1 and *k*, as defined in (36). The normalization function in (75), $p\left(y_{k},y_{k-1}|Y^{k-2}\right)$ is the consequence of the total probability theorem with reference to both models (m=1:2) under consideration at scan k−N, such that

(76) $$P\left\{M_{k-2}^{1}|Y^{k}\right\}+P\left\{M_{k-2}^{2}|Y^{k}\right\}=1.$$

In general, to obtain the smoothed mode probability for *j*th mode at fixed lag *N* using the measurement information till the current scan *k*, the following set of equations can be used,

(77) $$\begin{array}{c} P\left\{M_{k-N}^{j}|Y^{k}\right\}=\frac{\sum_{i_{1}=1}^{M}\sum_{i_{2}=1}^{M}\;..\;\sum_{i_{N}=1}^{M}\;\left[\;\begin{array}{c} \Delta_{k}\left(M^{i_{N}}\right)\Delta_{k-1}\left(M^{i_{N-1}}\right)..\Delta_{k-N+1}\;\left(M^{i_{1}}\right)\\ p_{ji_{1}}p_{i_{1}i_{2}}..p_{i_{N-1}}p_{i_{N}}P\left\{M_{k-N}^{j}|Y^{k-N}\right\} \end{array}\;\right]\;}{p\left(y_{k},y_{k-1},...,y_{k-N+1}|Y^{k-N}\right)} \end{array}\;,$$

where

(78) $$\begin{array}{c} p\left(y_{k},y_{k-1},...y_{k-N+1}|Y^{k-N}\right)=\sum_{m=1}^{M}p\left(y_{k},y_{k-1},...y_{k-N+1},M_{k-N}^{m}|Y^{k-N}\right).\; \end{array}$$

With the use of (77) and (78), one can find the smoothed mode probability at any past scan at fixed lag *N*.

**5.4: Merged Smoothed Target Trajectory State Update**

Each *j*th model smoothed state is probabilistically weighted and merged to calculate the smoothed target trajectory state at fixed lag *N* for the *j*th model. For this purpose, the smoothed target trajectory state ${\hat{x}}_{k-N|k}^{j}$ at fixed lag *N* from the augmented state smoothed target trajectory state (56) is used and weighted with the *j*th smoothed mode at fixed lag *N*, as calculated in (77), and merged over all the possible modes.

(79) $$\begin{array}{c} p\left(x_{k-N}|\chi_{k-N},Y^{k}\right)=\sum_{j=1}^{M}p\left(x_{k-N}^{j}|\chi_{k-N},Y^{k}\right)P\left\{M_{k-N}^{j}|Y^{k}\right\}\\ {\hat{x}}_{k-N|k}=\sum_{j=1}^{M}{\hat{x}}_{k-N|k}^{j}\mu_{k-N|k}\left(M^{j}\right) \end{array},$$

where all parameters are defined in the earlier sections.

## 4. Simulation Analysis

In this section, the performance of proposed algorithm is compared with different tracking algorithms under the same simulation conditions to achieve a fair analysis. A single target under maneuvering is considered in a 2-dimensional surveillance area with a dimension of 570m×270m. The target is assumed to follow two motion models: a CV model and a CA model. During the CV model, the target is assumed to be moving with a velocity of 17 m/s. In the CA model, the target is assumed to have an acceleration of $1.118\;{m/s}^{2}$.

**Figure 4:**
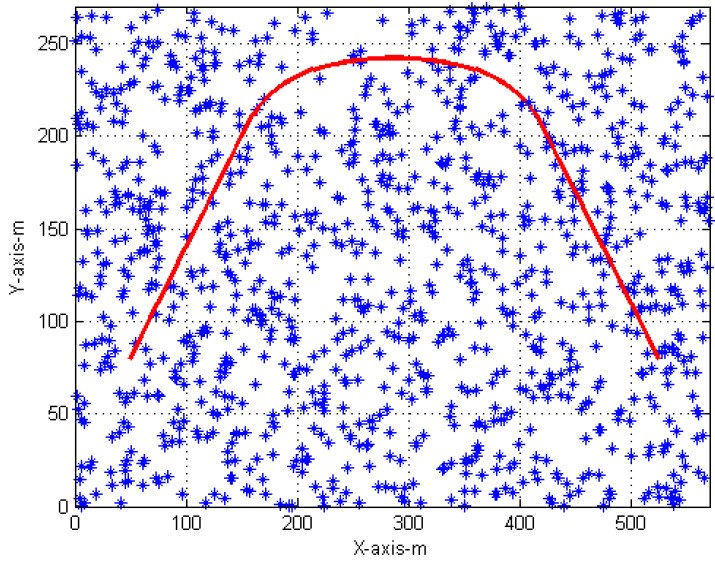
Simulation scenario (red: true target, blue: clutter).

As shown in Figure 4, the CV model is followed by the target in two separate scan intervals. The first target is in-between scans 1–21, and the second target is in-between scans 47–67. The CA model is followed by the target in-between scans 22–46. The initial position of the target is ${\left[50\;m,80\;m\right]}^{T}$ for the single target and the target is detected with a detection probability of $P_{d}=0.9$. The sampling time is 1 s. The total number of scans in a single run is 67 and 500 simulation runs are performed in this analysis. The clutter is uniformly distributed with a density of $10^{-4}\;m^{-2}$.

The root mean square error (RMSE), true track rate (TTR), and mode probabilities are presented in this section to compare the performance of the proposed algorithm with the other algorithms available in the literature, which also assumed the target existence state as an event [12].

**Figure 5:**
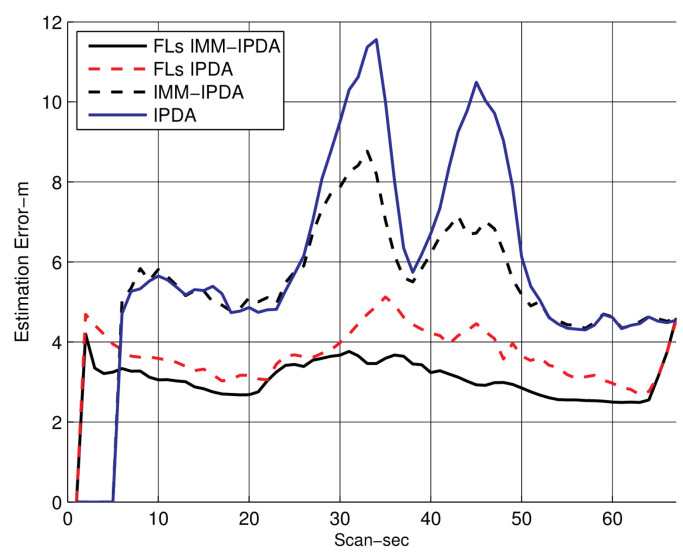
Root mean square error (the smoothing lag is 4).

**Figure 6:**
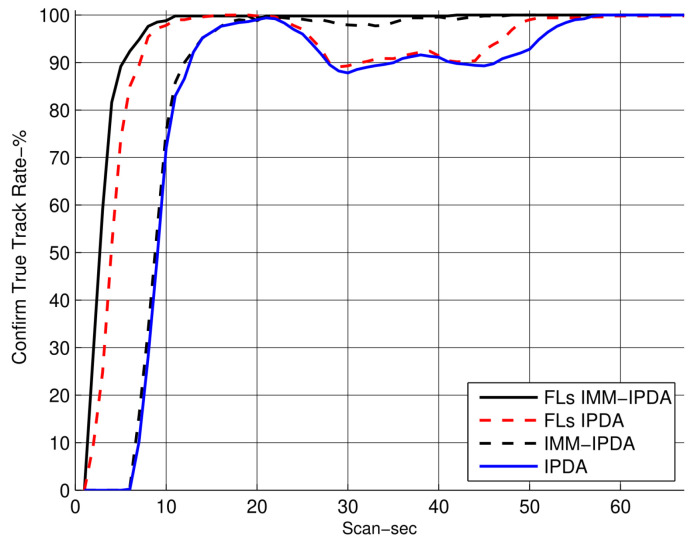
The confirmed true track rate (the smoothing lag is 4).

In Figure 5, the RMSE plots for different algorithms are compared with the proposed algorithm. The RMSE performance of the proposed algorithm is shown for the fixed lag N=4. It can be observed that the RMSE performance is improved as compared to the other tracking algorithms. In Figure 6, the CTTR for the proposed algorithm is plotted and it is compared with other different algorithms, which also compare the target existence as an event. The proposed FLs IMM-IPDA tracking algorithm confirms the true tracks much earlier compared to the other existing algorithms.The drop rate is almost negligible in the maneuvering phase compared to the other algorithms.

**Figure 7:**
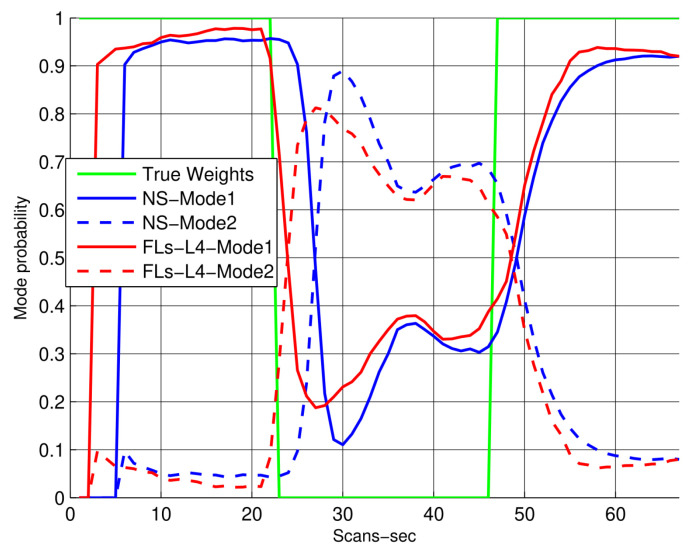
Mode probabilities (the smoothing lag is 4).

**Figure 8:**
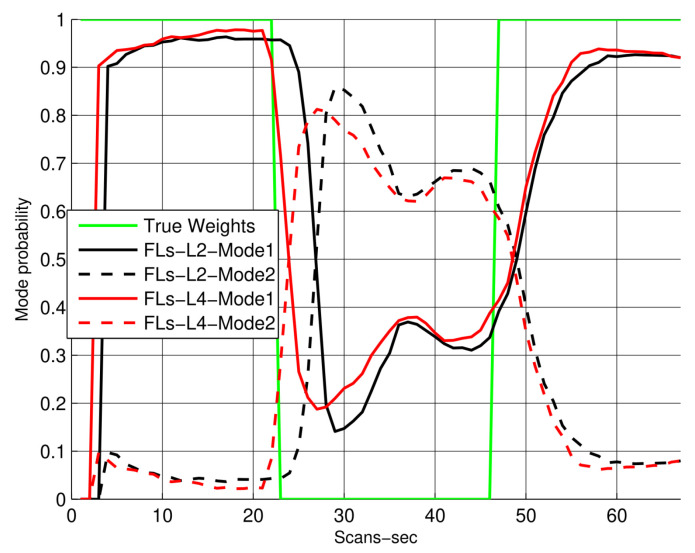
Mode probabilities for lag sizes 2 and 4.

In Figure 7, the mode probabilities for both the CV and CA models are compared among IPDA, IMM, and the proposed smoothing algorithm, which is used here to minimize the ambiguity in the figure (the legend shows NS:Non smoothing algorithm, which in this case is IMM-IPDA, and the proposed algorithm is labeled as FLs-L4, which implies fixed lag smoothing-Lag4). Based on the proposed formula used to obtain the smoothed mode probabilities, it can be observed that the proposed algorithm has shown a significant improvement in terms of mode decisions, and the mode switching is fast compared to other non-smoothing algorithms. Due to these smoothing probabilities, the overall target tracking is improved and its trajectory state estimation error is reduced, which is also evident in Figure 5. The performance of the proposed algorithm for different smoothing lags in terms of mode probabilities is also compared in Figure 8. It can be observed that the smoothing environment helps the algorithm to identify and correct the trajectory modes in a better and abrupt manner.

**Figure 9:**
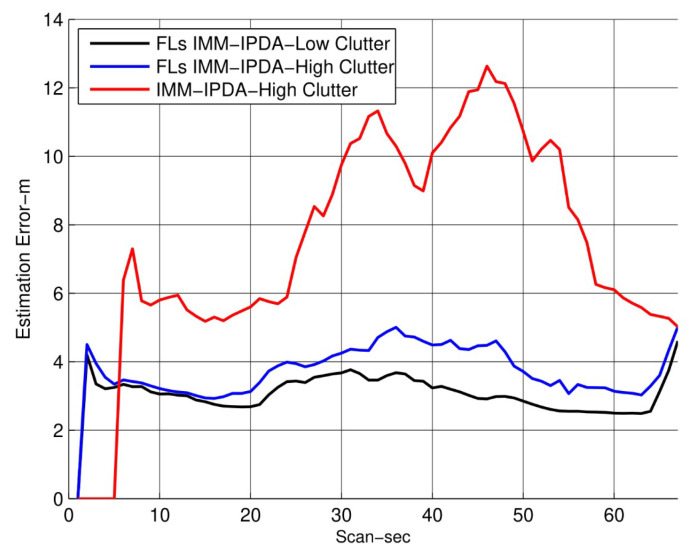
Root mean square error performance for clutter density: $2\times 10^{-4}\;m^{-2}$.

**Figure 10:**
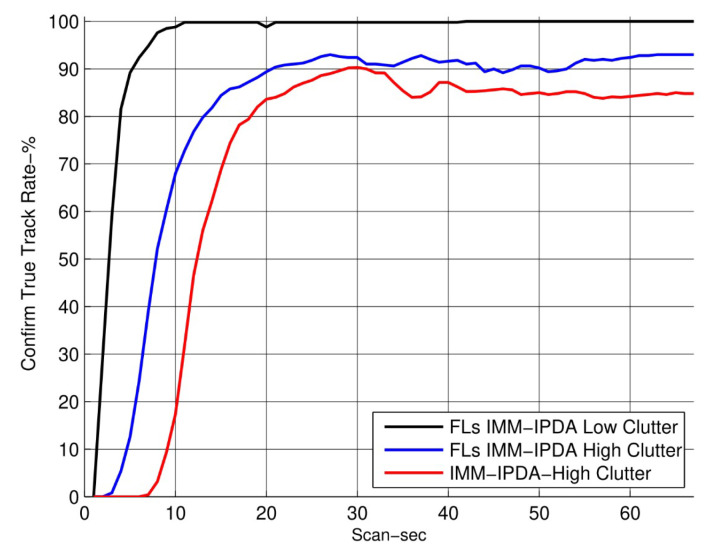
True track rate for clutter density: $2\times 10^{-4}\;m^{-2}$.

In addition to the above experiments, the results are compared for high clutter density in the same target trajectory environment. The results show the performance of the proposed algorithm in Figure 9 and Figure 10 for the RMSE, TTR, and mode probabilities. In this scenario, the clutter is twice as much, as compared to Figure 5, Figure 6, Figure 7 and Figure 8.

## 5. Conclusions

In this paper, a fixed lag smoothing technique is suggested to enhance the trajectory state and existence state of the target while it is being maneuvered in the presence of clutter. In addition, the proposed algorithm has provided a complete and generalize mathematical formula to smooth the target mode probabilities while it maneuvers during its motion in the presence of clutter.The target hybrid state and mode probabilities at fixed lag N are not smoothed using the standard IMM-IPDA technique due to the lack of a formal mathematical foundation. The suggested smoothing framework outperformed other methods in the literature, according to simulation findings for RMSE, CTTR, and mode probabilities.

## Data Availability

Data can be provided on demand.

## Appendix A

### Appendix A.1. Joint Mode Transition Probabilities

The joint mode transition probabilities in (65), (68), (71), and (74) are derived as

(A1) $$\begin{array}{c} P_{111}=P\left\{M_{k-2}^{1},M_{k-1}^{1},M_{k}^{1}|Y^{k-2}\right\}\\ P_{111}\;=P\left\{M_{k}^{1}|M_{k-1}^{1},M_{k-2}^{1},Y^{k-2}\right\}P\left\{M_{k-1}^{1}|M_{k-2}^{1},Y^{k-2}\right\}P\left\{M_{k-2}^{1}|Y^{k-2}\right\},\\ \;\;\;\;\;\;\;\;\;=P\left\{M_{k}^{1}|M_{k-1}^{1}\right\}P\left\{M_{k-1}^{1}|M_{k-2}^{1}\right\}P\left\{M_{k-2}^{1}|Y^{k-2}\right\},\\ \;\;\;\;\;\;\;\;\;=p_{11}^{2}P\left\{M_{k-2}^{1}|Y^{k-2}\right\}, \end{array}$$

(A2) $$\begin{array}{c} P_{112}=P\left\{M_{k-2}^{1},M_{k-1}^{1},M_{k}^{2}|Y^{k-2}\right\}\\ P_{112}\;=P\left\{M_{k}^{2}|M_{k-1}^{1},M_{k-2}^{1},Y^{k-2}\right\}P\left\{M_{k-1}^{1}|M_{k-2}^{1},Y^{k-2}\right\}P\left\{M_{k-2}^{1}|Y^{k-2}\right\}\\ \;\;\;\;\;\;\;\;\;\;\;=P\left\{M_{k}^{2}|M_{k-1}^{1}\right\}P\left\{M_{k-1}^{1}|M_{k-2}^{1}\right\}P\left\{M_{k-2}^{1}|Y^{k-2}\right\}\\ \;\;\;\;\;\;\;\;\;\;\;\;=p_{12}p_{11}P\left\{M_{k-2}^{1}|Y^{k-2}\right\} \end{array}$$

(A3) $$\begin{array}{c} P_{121}=P\left\{M_{k-2}^{1},M_{k-1}^{2},M_{k}^{1}|Y^{k-2}\right\}\\ P_{121}\;=P\left\{M_{k}^{1}|M_{k-1}^{2},M_{k-2}^{1},Y^{k-2}\right\}P\left\{M_{k-1}^{2}|M_{k-2}^{1},Y^{k-2}\right\}P\left\{M_{k-2}^{1}|Y^{k-2}\right\}\\ \;\;\;\;\;\;\;\;=P\left\{M_{k}^{1}|M_{k-1}^{2}\right\}P\left\{M_{k-1}^{2}|M_{k-2}^{1}\right\}P\left\{M_{k-2}^{1}|Y^{k-2}\right\}\\ \;\;\;\;\;\;\;\;=p_{21}p_{12}P\left\{M_{k-2}^{1}|Y^{k-2}\right\} \end{array}$$

(A4) $$\begin{array}{c} P_{122}=P\left\{M_{k-2}^{1},M_{k-1}^{2},M_{k}^{2}|Y^{k-2}\right\}\\ P_{122}\;=P\left\{M_{k}^{2}|M_{k-1}^{2},M_{k-2}^{1},Y^{k-2}\right\}P\left\{M_{k-1}^{2}|M_{k-2}^{1},\;Y^{k-2}\right\}P\left\{M_{k-2}^{1}|Y^{k-2}\right\}\\ \;\;\;\;\;\;\;\;=P\left\{M_{k}^{2}|M_{k-1}^{2}\right\}P\left\{M_{k-1}^{2}|M_{k-2}^{1}\right\}P\left\{M_{k-2}^{1}|Y^{k-2}\right\}\\ \;\;\;\;\;\;\;\;=p_{22}p_{12}P\left\{M_{k-2}^{1}|Y^{k-2}\right\} \end{array}$$
